# Biological function of *Klebsiella variicola* and its effect on the rhizosphere soil of maize seedlings

**DOI:** 10.7717/peerj.9894

**Published:** 2020-09-16

**Authors:** Lijuan Yang, Kejun Yang

**Affiliations:** 1 College of Agronomy, Heilongjiang Bayi Agricultural University, Daqing, Heilongjiang, People’s Republic of China; 2 Key Laboratory of Crop Germplasm Improvement and Cultivation in Cold Regions of Education Department, Daqing, Heilongjiang, People’s Republic of China; 3 College of Graduate, Heilongjiang Bayi Agricultural University, Daqing, Heilongjiang, People’s Republic of China

**Keywords:** *Klebsiella variicola*, Saline-alkali stress, Rhizosphere microenvironment, Maize

## Abstract

**Background:**

Deterioration of the ecological environment in recent years has led to increasing soil salinization, which severely affects the cultivation of agricultural crops. While research has focused on improving soil environment through the application of pollution-free microbial fertilizers, there are relatively few plant growth-promoting bacteria suitable for saline-alkali environments. Although *Klebsiella variicola* can adapt to saline-alkali environments to successfully colonize rhizosphere microenvironments, only a few studies have investigated its role in promoting crop growth. Its effect on the crop rhizosphere soil microenvironment is especially unclear.

**Methods:**

In this study, the biological function of *K. variicola* and its colonization in maize seedling rhizosphere soil were studied in detail through selective media and ultraviolet spectrophotometry. The effects of *K. variicola* on the rhizosphere soil microenvironment and the growth of maize seedlings in saline-alkali and neutral soils were systematically analysed using the colorimetric method, the potassium dichromate volumetric method, and the diffusion absorption method.

**Results:**

Our results showed that *K. variicola* played a role in indole acetic acid, acetoin, ammonia, phosphorus, and potassium production, as well as in nitrogen fixation. A high level of colonization was observed in the rhizosphere soil of maize seedlings. Following the application of *K. variicola* in neutral and saline-alkali soils, the nutrient composition of rhizosphere soil of maize seedlings increased in varying degrees, more notably in saline-alkali soil. The content of organic matter, alkali-hydrolysable nitrogen, available phosphorus, available potassium, alkaline phosphatase, sucrase, urease, and catalase increased by 64.22%, 117.39%, 175.64%, 28.63%, 146.08%, 76.77%, 86.60%, and 45.29%, respectively, insaline-alkalisoil.

**Conclusion:**

*K.variicola*, therefore, performed a variety of biological functions to promote the growth of maize seedlings and effectively improve the level of soil nutrients and enzymes in the rhizosphere of maize seedlings, undersaline-alkali stress conditions. It played an important role in enhancing the rhizosphere microenvironment of maize seedlings under saline-alkali stress.

## Introduction

Salty soil is widely distributed on earth, with more than 950 million hectares of saline-alkaline land area, distributed globally according to the statistics (Li & Liu, 2018). Soil salinization has become a global ecological issue, and for global agriculture, there is an urgent need to solve the problem. Saline soil is a serious threat to agricultural crop growth and development, and it gradually becomes the main stressor. Songnen Plain, located in northeast China, is a region with strongly saline-alkali soil, and the affected area continues to grow every year (Yang & Wang, 2015). The main components of saline-alkali soil in Heilongjiang province are Na_2_CO_3_ and NaHCO_3_, which are typical soda-saline-alkali soil components. The formation of saline-alkali soil is mostly related to the accumulation of carbonate content in the soil, leading to a very severe degree of alkalinisation and almost no plant life in severely affected areas (Zhang, Wang & Yang, 2015). Soil salinization reduces a soil’s own nutrient levels, destroys good aggregate structure in the soil, and significantly affects crop growth and development (Gupta & Huang, 2014; Yu, Qian & Wei, 2019).

Corn, a salty crop, is widely grown in China. According to the China Statistical Yearbook, China was the country with the highest total sown area and yield of corn in 2019. The 2019 China statistical yearbook showed that corn planting density resulted in it being among the most abundant crops in China (Luo et al., 2019). Regions with greater corn production also receive more attention and funding. Heilongjiang province is one of the main grain-producing areas in China, and corn as the first major crops in Heilongjiang province, covers a crop area of 5.821 million hm^2^, which yields 35.404 million t/year (Wang, 2019). High and stable corn yield in the region is directly related to national economic development. However, maize especially maize seedlings are sensitive to salt and pH, thus saline-alkali soil seriously affects the growth and development of maize seedlings (Li, Jiang & Bie, 2009). Currently, researchers are trying to determine how can the impact of saline soil on the growth of maize be alleviated, how can the rhizosphere soil microenvironment of maize seedlings be improved, and how can the saline-alkali resistance ability of maize seedlings be improved. The rational utilization of saline soil resources is of great significance to the sustainable development of agriculture in saline and alkaline areas in Heilongjiang Province of China and the world at large.

In recent years, the use of microbial agents to improve crop growth and soil improvement has attracted much attention. The application of microbial fertilizers can improve the microenvironment of crop rhizosphere soil, and alleviate the damage caused by saline-alkali stress to the crops, in order to improve saline-alkali soils (Ke et al., 2015; Zhang et al., 2017). Microorganisms that improve the maize rhizosphere soil microenvironment are currently mainly concentrated in a few genera, such as Bacillus and Pseudomonas (Li et al., 2015; Zhang et al., 2016), and the effect of their application in saline-alkali environments is not ideal (Jiang et al., 2019). *K. variicola* is a potential candidate as a plant growth-promoting bacterium, and it has played a very important role in agricultural production. At present, most studies on the role of Klebsiella have only been conducted on rice and soybean crops (Liu, 2018), where research has shown that Klebsiella can promote the growth of rice, and increase its yield by 28% (Sangabriel et al., 2015). Other researchers found that klebsiella Sneb YK can induce production of ribulose-1,5-bishosphate carboxylase small subunits, a protein involved in photosynthesis; and inhibit the activity of soybean pesticide residues. In this way, Klebsiella can alleviate the harmful effects of atrazine on soybean plants and ensure they grow normally (Chen et al., 2014).

However, there are few studies which have investigated Klebsiella’s effects on crop rhizosphere soil, mainly in terms of the effects of pesticides on soil degradation. It was reported that Klebsiella pneumoniae can degrade endosulfan pesticides (Abraham & Silambarasan, 2014), chlorpyrifos, trichlorfon (Nan, 2013), herbicides azo dye methyl red (Cui, 2012), and others. Studies have shown that Klebsiella can colonize tomato roots, and the colonization concentration in the soil was 1.1 × 10^6^ cfu/g after being applied for 28 days (Wang, 2006). These studies have proved that Klebsiella could colonize in the rhizosphere soil of plants for a long time, which laid a foundation for the study of Klebsiella to improve the microenvironment of rhizosphere soil. However, the effects of Klebsiella on soil nutrients and enzymes in the rhizosphere remain unclear. There are no reports on how to improve the rhizosphere soil microenvironment of maize seedlings and alleviate the effects of saline-alkali stress on the latter, especially in saline-alkali soil. The objective of this study was to determine the mechanisms that enable *K. variicola* to promote the growth of maize seedlings and improve their rhizosphere microenvironment, which plays an important role in alleviating the damage caused by saline-alkali stress in plants.

## Materials & Methods

### Bacterial strain, soil, and plant material

*K. variicola* (CGMCC1. 15640) was acquired from the China General Microbial Species Preservation and Management Center. Natural saline-alkali soil (pH 9.2, Na^+^ content: 0.906 g kg^−1^) was obtained from Daqing City, Heilongjiang Province. Neutral soil (pH 7.4, Na^+^ content: 0.628 g kg^−1^) was obtained from Zhaodong County, Heilongjiang Province. Corn seed was Xianyu 335, and was purchased from Heilongjiang Fuzun Agricultural Integrated Service Chain Co. Ltd.

### Instruments

Autoclavous steam sterilizer (Hitachi, Japan), super clean workbench (Boke, Beijing), constant temperature oscillating culture shaker (Zhicheng, Shanghai), light incubator (Shanghai Boxun, Shanghai), high speed frozen centrifuge (Kecheng Hunan).

### Main drugs

Sucrose, NaCl, K_2_HPO_4_ ⋅ 3H_2_O, CaCO_3_, MgSO_4_ ⋅7H_2_O, Agar, naphthol, creatine, acetoin, toluene, benzenedisodium phosphate, 4-aminoantiimidoline, potassium ferricyanide, purchased from Daqing City Salto District Yineng Materials Sales Office

### Induction and colonization of rifampicin (Rif) resistant strains

The colonization of *K. variicola* was determined using the antibiotic labelling method (Gao et al., 2020). Rifampicin resistance was induced in the bacterial strain by increasing the concentration of rifampicin to 300 µg mL^−1^, using a concentration gradient set up at 50, 100, 150, 200, 250, and 300 µg mL^−1^. The strain was allowed to grow normally in LB medium containing the lowest concentration of rifampicin, and then transferred to the next higher concentration for induction, until the strain was able grow normally at a concentration of 300 µg mL^−1^ rifampicin.

Determination of genetic stability of rifampicin resistance: Following rifampicin resistance induction, the genetic stability of the antibiotic resistance was determined by transferring rifampicin resistant strains to LB plates without rifampicin for 5 generations. The strains were then transferred back to LB plates containing 300 µg mL^−1^ rifampicin to detect its genetic stability. Normal growing bacteria were identified as positive, and vice versa.

Determination of strain colonization: *K. variicola* was inoculated using the root irrigation method, whereby each pot was inoculated with 100 mL of Rif-resistant bacterial suspension (with the concentration of *K. variicola* at 1 × 10^8^ cfu mL^−1^), and pot culture was carried out. At 0, 3, 6, 9, 12, and 15 d after inoculation, 1 g of soil was collected from the rhizosphere of plant seedlings and diluted with sterile water. Tenfold gradient dilution was carried out, and the diluted bacterial suspension was coated on LB solid plate with colony counting method. After 48 h, the bacterial colonies on the plate were counted. The colony number of labelled strains per gram of soil was determined. Rifampicin-resistant strains were isolated using LB selective medium, prepared by adding 300 µg mL^−1^ of rifampicin solution to LB medium.

### Preparation of bacterial suspension

*K. variicola* was inoculated in LB liquid medium, then the culture flasks were put in a shaker incubator and incubated at 30 °C and 180 rpm for 24 h. The bacterial suspension was then centrifuged at 6,000 × *g* for 15 min, and the supernatant was discarded. The precipitate was washed with aseptic distilled water and re-suspended. This process was repeated three times. It was then diluted to 1 × 10^2^ cfu mL^−1^, 1 × 10^4^ cfu mL^−1^, and 1 × 10^6^ cfu mL^−1^, according to the requirements of the experiment.

### Maize sprouting treatment

Xianyu 335 corn seeds of similar size and showing no damage on the surface were selected, soaked, and disinfected for 10 min using a 10% sodium hypochlorite solution. Seeds were then washed with sterile water more than five times until there was no obvious trace of sodium hypochlorite. The sterilized seeds were soaked in sterile water and transferred to an incubator maintained at 25 °C for 6 h, before discarding the sterile water. A piece of filter paper was spread across the bottom of the germination box and sterile water was evenly added until the filter paper was slightly soaked. Imbibed seeds were spread out evenly in the germination box, with 20 seeds in each box, and a piece of filter paper evenly coated with 3 mL sterile water was spread across the seeds. The germination box was covered with a lid and placed in a 25 °C incubator for 24 h, in order to accelerate germination in darkness.

### Pot-based experiment

Potted soil was thoroughly air-dried and passed through a 2-mm sieve. Each pot (10 cm length × 10 cm width × 12 cm height) was loaded with 200 g of soil. Corn seeds with bud lengths of approximately 1 cm (0. 9 cm –1. 1 cm) were planted in pots filled with soil. Five seeds were evenly sown in each pot, which was then topped with 100 g more of soil. Bacterial suspensions (100 mL) at different concentrations (1 × 10^2^ cfu/ml, 1 × 10^4^ cfu/ml, 1 × 10^6^ cfu/ml, 1 × 10^8^ cfu/ml) were poured into each pot. The pots were cultured in a light incubator for 15 d, and 10 mL of sterile water was added to each pot every 48 h, for the duration of the culture period. The control group was set up without the addition of a bacterial suspension. The culture conditions were as follows: the day and night temperatures were set at 25 °C and 20 °C, respectively, light was set at a 12:12 h light:dark cycle, and the humidity was at 60%–80%.

### Determination of the biological function of *K. variicola*

Nitrogen fixation ability was determined by inoculating activated *K. variicola* colonies on a nitrogen-free solid medium [sucrose (10 g), NaCl (0.12 g), K_2_HPO_4_ ⋅3H_2_O (0.5 g), CaCO_3_ (1 g), MgSO_4_ ⋅7H_2_O (0.2 g), Agar (20 g), distilled water (1,000 ml), pH 7.2]. The presence or absence of *K. variicola* growth was observed to determine whether the bacteria possessed the ability to fix nitrogen. (Singh et al. 2015)

*K. variicola* was inoculated into 250-mL flasks containing nitrogen-free liquid medium, and a no inoculation (CK) condition was set up as the control. Each treatment was repeated 3 times at 30 °C, and 180 rpm, and flasks were cultured for 7 d. The culture medium was then divided into two parts, one of which was centrifuged at 6,000 × *g* for 15 min, and the supernatant was collected. The centrifuged supernatant and corresponding uncentrifuged sample were diluted 10x with ammonia-free water. Determination of soluble nitrogen concentration in the fermentation broth was by potassium persulfate oxidation-UV spectrophotometry (corresponding to determination of total nitrogen in water; GB-11894-89).

Determination of phosphorus solubilizing ability: *K. variicola* was inoculated on inorganic phosphorus and organophosphorus plates. Inorganic phosphorus medium (Ji, Gururani & Chun, 2014) consisted of glucose (10 g), Ca_3_(PO_4_)_2_ (5 g), MgCl_2_ ⋅6H_2_O (5 g), MgSO_4_ ⋅7H_2_O (0.25 g), KCl (0.2 g), (NH_4_)_2_SO_4_ (0.1 g), Agar (20 g), distilled water (1,000 mL), at pH 7.0. Among these chemicals, Ca_3_(PO_4_)_2_ was sterilized separately, and then mixed with other components in the medium once the temperature had reduced to 70 °C. Organophosphorus medium (Wang, 2010) contained glucose (10 g), (NH_4_)_2_SO_4_ (0.5 g), NaCl (0.3 g), FeSO_4_ ⋅7H_2_O (0.03 g), MnSO_4_ ⋅4H_2_O (0.03 g), CaCO_3_ (5 g), yeast extract (0.4 g), Agar (20 g), lecithin (0.2 g), distilled water (1,000 mL), at pH 7.0. Following bacterial culture at 30 °C for 7 d, the occurrence of a transparent phosphorus solubilizing circle on either the inorganic or organic phosphorus medium was interpreted as the bacteria having the capability of dissolving inorganic or organic phosphorus, respectively. The ability to dissolve phosphorus was gauged according to the ratio of the radius of the phosphorus dissolving circle to the radius of the colony (Ke et al., 2015; Zeng, Wen & Wu, 2016). Dissolved inorganic phosphorus by *K. variicola* was quantitatively detected using the molybdenum-antimony colorimetric method (Lang, 2014).

### Determination of potassium dissolving ability

The soluble potassium content in the *K. variicola* fermentation broth was determined using sodium tetraphenylborate (Ran, Ma & Meng, 2009). The standard curve was drawn according to the absorbance value and the content of potassium in solution at 420 nm. The soluble potassium content in the *K. variicola* fermentation broth was determined from the standard curve.

### Determination of ammonia production capacity

Activated *K. variicola* was inoculated into a test tube containing 10 mL of protein water, according to the method described Singh, Jha & Jha (2015). Following incubation at 30 °C for 48 h, 0.5 mL of reagent was added to each test tube, and uninoculated medium was used as a blank control. If the strain appeared to be brown to yellow in colour, following the addition of Nessler’s reagent, it was considered able to produce ammonia. Ammonia production by *K. variicola* was quantitatively detected using Nessler’s reagent colorimetric method (Zheng, 2006).

The ability of *K. variicola* to produce indole acetic acid was determined according to the method described by Wang, Yuan & Zhao (2010). L-tryptophan, the precursor of indole-acetic acid, was added to the nitrogen-containing medium, which was inoculated with *K. variicola* and cultured at 180 rpm at 30 °C for 3 d. The fermentation broth containing *K. variicola* was centrifuged at 6,000 × *g* for 15 min, and the collected supernatant was mixed with 50 µL of 10 mM phosphoric acid, followed by the addition of 2 mL of Salkowski chromogenic solution (0. 5 M FeCl_3_ ⋅6H_2_O and 98 mL of 35% HClO_4_, mixed evenly). The colour changes were observed after incubation for 30 min in the dark, at 30 °C. The quantitative determination of indole acetic acid production by *K. variicola* was deduced from the absorbance value of discoloration solution at 530 nm. The concentration of indole acetic acid produced by *K. variicola* was calculated from the standard curve of indole acetic acid.

### Determination of the ability to produce acetoin

*K. variicola* was cultured for 24 h in V-P selective medium [glucose (5 g), peptone (5 g), K_2_HPO_4_ (5 g), distilled water (1,000 mL)], and 1 mL of the supernatant was added to creatine chromogenic solution (0.6 mL of 5% *α*-naphthol, 0.6 mL of 0.5% creatine, and 0.2 mL of 40% KOH). If the solution turned red, the strain produced acetoin. A standard curve was drawn from colour reaction with a range of concentrations of acetoin solution, and the concentration of acetoin produced by *K. variicola* was quantitatively detected (Liu, 2018).

### Determination of soil nutrients and enzyme activities

To determine alkaline phosphatase activity, 5 g of soil and 0.5 mL toluene was added. The components were allowed to mix for 15 min, and 20 mL of 0.5% disodium benzene phosphate solution was added. The components were mixed well and placed in an incubator maintained at 37 °C for 2 h. After the culture period, 5 mL of filtrate was transferred into a 50-mL volumetric flask, followed by 0.25 mL of buffer solution, 0.5 mL of 4-aminoantipyrine solution, and 0.5 mL of potassium ferricyanide solution. Once the colour was stable (after approximately 15 min), the optical density was measured at a wavelength of 510 nm (Guan, 1986; Zhou et al., 2016).

### Determination of urease activity

A soil sample (5 g) was treated with 1 mL toluene for 15 min. A 10% urea solution (10 ml; pH 6.7) and citrate buffer (20 mL) was added to the flask, and the mixture was incubated at 37 °C for 24 h. The collected filtrate (3 mL) was then mixed with an addition 20 mL of distilled water, 4 mL of phenol sodium solution, and 3 mL of sodium hypochlorite solution. Colour developed after 20 min, and the volume was adjusted. The spectrophotometer was used, within 1 h, to compare the absorbance at a wavelength of 578 nm (Bao, 2000).

### Determination of catalase activity

A soil sample (2 g) with the addition of 40 mL of distilled water and 5 mL of 0.3% H_2_O_2_. The flask placed in an oscillator (120 rpm) was maintained at 30 °C constant temperature, for 20 min. Immediately after, 1 mL of saturated aluminium potassium alum was added to the flask, and the contents were immediately filtered into a triangular flask containing 5 mL of 1.5 mol L^−1^ sulfuric acid solution. The filter was dried, and absorbance of the filtrate was determined directly at 240 nm using a 1-cm quartz colorimetric plate. The test results were expressed as the difference between the control and the test (Yang et al., 2011).

### Determination of sucrase activity

A soil sample (2 g) injected with 15 mL of 8% sucrose solution, 5 mL of phosphate buffer solution (pH 5.5), and 0.25 mL of toluene. The mixture was incubated at 37 °C for 24 h. The filtrate (1 mL) and 3 mL of 3, 5-dinitrosalicylic acid were mixed. The mixture was heated in a boiling water bath for 5 min and subjected to colorimetric analysis at 508 nm wavelength on a spectrophotometer (Li, Wang & Hong, 2010).

### Determination of organic matter content

A sample of air-dried soil (0.3 g) was weighed. A standard solution of 0.8 mol L^−1^ K_2_Cr_2_O_7_ (5 mL) was added, followed by injection with 5 mL of concentrated sulfuric acid. The test tube was heated in an oil bath, with the temperature controlled at 170 °C–−180 °C. The mixture was heated for 5 min. The test tube was then removed Three drops of o-phenanthroline indicator was added to the bottle, and the mixture was titrated with a 0.2 mol L^−1^ FeSO_4_ solution. The end point occurred when the solution colour changed from green to brownish red (Lu, 1999).

### Determination of alkali-hydrolysable nitrogen content

An air-dried soil sample (2 g) was weighed and spread evenly in the outer chamber of a diffusion dish. A 2% boric acid mixed indicator solution (2 mL) was added to the diffusion dish chamber. A 1.8 mol L^−1^ sodium hydroxide solution (10 mL) was added to the diffusion dish outer chamber, and the side of the cover glass slide was immediately tightly pushed back. The diffusion dish was transferred to an oven maintained at 40 °C, for 24 h, to allow alkaline hydrolysis and diffusion to take place. The amount of ammonia absorbed by indoor boric acid was titrated with 0.01 mol L^−1^ hydrochloric acid, with a colour change from blue to reddish (Shi et al., 2017).

### Determination of available phosphorus content

An air-dried soil sample (2.5 g) and 50 mL of 0.5 mol L^−1^ NaHCO_3_ solution was added. The mixture was shaken for 30 min. The filtrate (10 mL) was then absorbed in a 25-mL capacity flask, and 5 mL of molybdenum and antimony antichromogenic agent was added. The mixture was incubated at 37 °C for 30 min, before being subjected to colorimetric analysis at 880 nm (Xiao et al., 2012).

### Determination of available potassium content

An air-dried soil sample (2 g) was placed in a 150-mL flask, and 20 mL of 1 mol L^−1^ NH_4_OAc solution was added. The mixture was shaken at 25 °C for 30 min, filtered, and the filtrate was analysed on a flame photometer at 766.5 nm (Meng et al., 2014).

### Statistical analysis

One-way analysis of variance tests were conducted with SPSS 21.0 software (SPSS Inc., Chicago, IL, USA). Duncan’s test method was employed for multiple comparisons and analysis of the differences; significance was set as *P* < 0.05. All data in the tables are average values of triplicate or more repetitions.

## Results

### Biological function of *K. variicola*

#### Nitrogen fixation capacity of *K. variicola*

**Figure 1:**
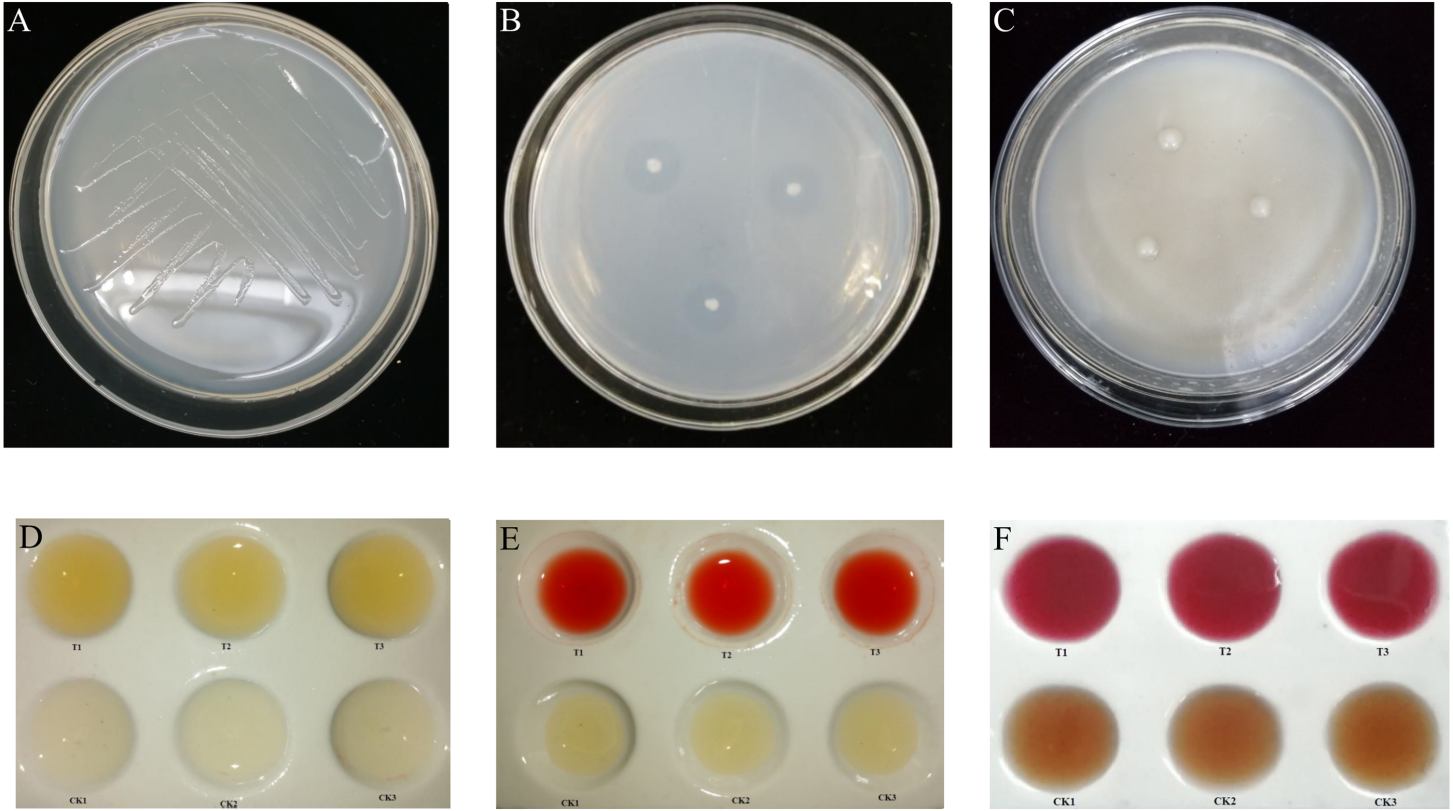
Biological function of *K. variicola*. (A) Growth of *Klebsiella variicola* in a nitrogen free medium. (B) Growth of *Klebsiella variicola* in an inorganic phosphorus medium. (C) Growth of *Klebsiella variicola* in an organophosphorus medium. (D) Determination of ammonia production capacity of *Klebsiella variicola*. (E) Determination of indole-acetic-acid production capacity of Klebsiella variicola. (F) Determination of acetoin production ability of *Klebsiella variicola*.

Following culture on a nitrogen-free solid medium for 72 h, *K. variicola* grew well, as shown in Fig. 1A, indicating the bacteria’s ability to perform nitrogen fixation. Following bacterial culture in nitrogen-free liquid medium for 15 d, the concentration of soluble nitrogen in the fermentation broth was determined by potassium persulfate oxidation-UV spectrophotometry. The results showed that the concentration of soluble nitrogen in fermentation broth treated with *K. variicola* was 6.5 mg L^−1^, and that the amount of nitrogen fixed by the bacteria in each litre of culture medium was 9.2 mg, with the total amount of nitrogen obtained via fixation being 15.7 mg L^−1^. This indicated that *K. variicola* possessed the ability to effectively fix nitrogen.

#### Phosphorus solubilizing ability of *K. variicola*

The inoculation of *K. variicola* on an inorganic phosphorus solid medium produced obvious phosphorus-dissolving circles around the bacterial colonies after 3 d of culture, as shown in Fig. 1B. The average ratio of D/d was 4.438 ± 0.390. Using the molybdenum-antimony colorimetric method, the standard curve was drawn, according to the absorption values of phosphorus-molybdenum blue formed from standard phosphate of different gradient concentrations, measured at 882 nm. The soluble phosphorus content in the inorganic phosphorus fermentation broth with *K. variicola* was 62.16 ± 1.27 mg L^−1^. Inoculation of *K. variicola* on organophosphorus solid medium produced no obvious phosphorus-solubilizing circles around the colonies after 6 d of culture (Fig. 1C). These results indicated that *K. variicola* possessed the ability to dissolve inorganic phosphorus but failed to dissolve organic phosphorus.

#### Potassium-solubilizing capacity of *K. variicola*

*K. variicola* was inoculated in potassium solution liquid medium and cultured for 5 d. Using sodium tetraphenylboron, the standard curve was drawn, according to the absorbance of standard potassium at different gradient concentrations, at 420 nm. The content of soluble potassium in the fermentation broth with *K. variicola* was 31.38 ± 1.03 mg L^−1^. The results showed that *K. variicola* possessed the ability to dissolve potassium.

#### Ammonia production capacity of *K. variicola*

Following the inoculation and culture of *K. variicola* in protein water for 3 d, a notable yellow precipitation was produced with the addition of Nessler’s reagent to the fermentation broth. The uninoculated CK group, however, failed to produce a chromogenic reaction, indicating that *K. variicola* can produce ammonia (Fig. 1D). The standard curve was drawn, according to the absorbance value of the yellowish-brown complex formed at different concentrations of the ammonia-nitrogen solution, together with Nessler’s reagent at their corresponding solution concentrations, at 420 nm. The ammonia-producing activity of *K. variicola* was 56.25 ± 3.41 mg L^−1^.

#### Indoleacetic acid (IAA) production capacity of *K. variicola*

Following the inoculation and culture of *K. variicola* in liquid medium containing L-tryptophan for 3 d, Salkowski chromogenic solution was added to the top layer of supernatant. An obvious pink colour was observed after 30 min, consistent with the colour reaction of indole acetic acid. No colour was observed in the CK group, which was not inoculated with *K. variicola*, indicating that *K. variicola* has the ability to produce indole acetic acid (Fig. 1E). Based on to the absorbance measured from the chromogenic solution at 530 nm, and on the concentration of the IAA standard solution, the standard curve was drawn. The concentration of IAA produced by *K. variicola* was calculated to be 399.43 ± 11.56 mg L^−1^.

#### Indole acetoin production ability of *K. variicola*

A creatine chromogenic solution was added to *K. variicola* cultured in a V-P selective medium for 48 h. Results showed that the fermentation broth containing *K. variicola* turned pink after the addition of the creatine chromogenic solution, while no colour change was observed in the culture medium of the blank control group (Fig. 1F), indicating that *K. variicola* can produce acetoin. Using different concentrations of acetoin standard solution from the colour reaction to draw the standard curve, the acetoin production capacity of *K. variicola* was 152.88 ± 13.56 mg L^−1^.

#### Colonization of *K. variicola* in soil

**Figure 2:**
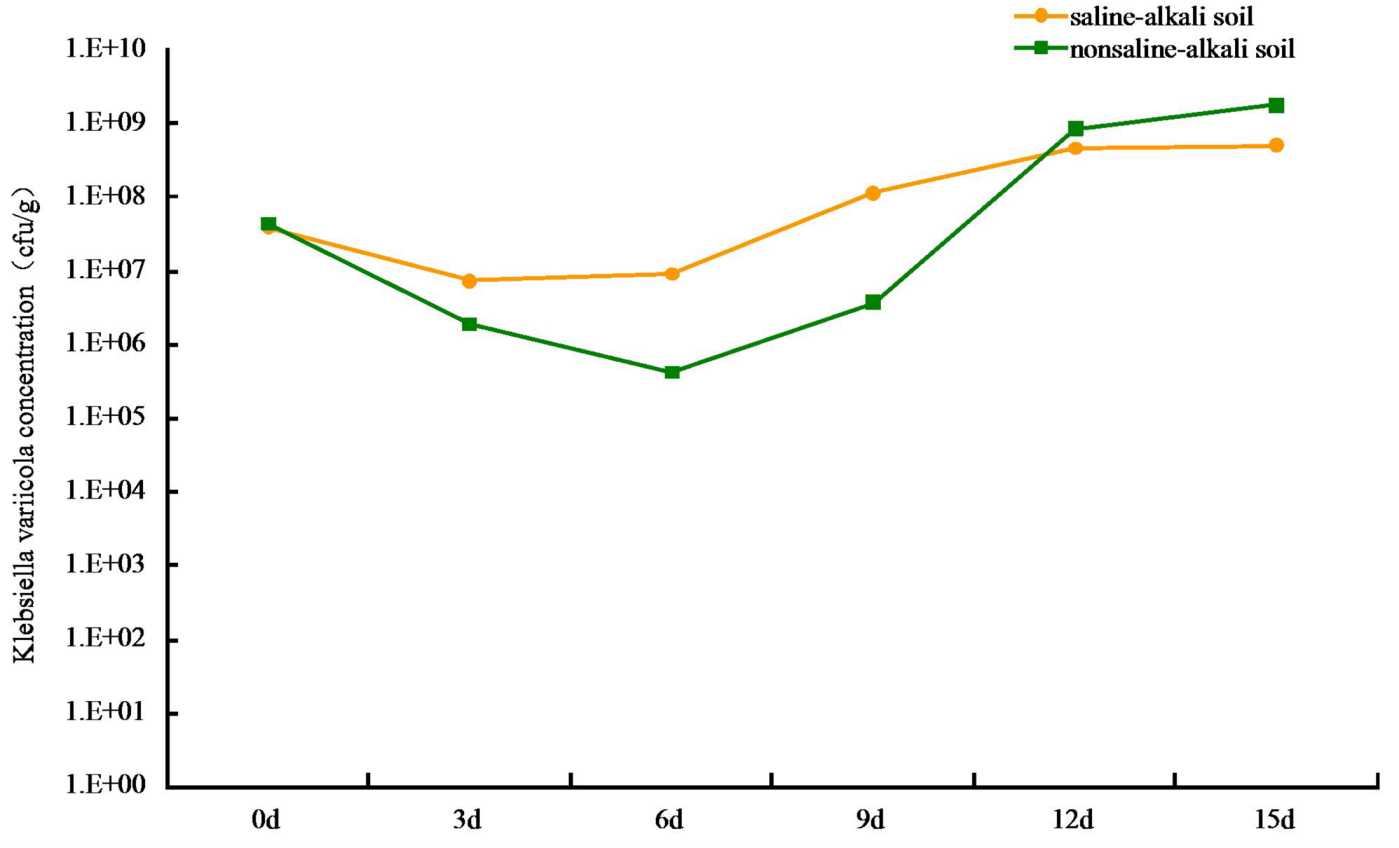
Colonization of *Klebsiella variicola* in soil.

The colonization of *K. variicola* in both saline-alkaline and neutral soils produced an initial decrease, followed by an increase in cfu number. The lowest level of colonization in saline-alkali soil was at 7.35 × 10^6^ cfu g^−1^, when the treatment was applied for 3 d. The process appeared to stabilize after 12 d, and the colonization reached 4.68 × 10^8^ cfu g^−1^ (Fig. 2). This may be owing to the competition from other microorganisms present in the saline-alkali soil and to additional feeding by protozoa, which resulted in a decrease in the number of *K. variicola* colonies formed in the early stages of treatment. As time progressed, the colonization *K. variicola* gradually increased, and the number of bacterial colonies appeared to reach an equilibrium with the external environment after 12 d. Application of the bacteria in neutral soil for 6 d produced the lowest level of colonization at 4.27 × 10^5^ cfu g^−1^. The level of colonization subsequently gradually increased and reached 1.78 × 10^9^ cfu g^−1^ on the 15th day. The types of species, as well as number of microorganisms and protozoa present in neutral soil, were more than those present in saline-alkali soil. This created a more intense competition and supplementary feeding effect of *K. variicola* in neutral soil than in saline-alkali soil, causing the colonization number to be reduced to 4.27 × 10^5^ cfu g^−1^ in the early stages of application. As the nutrient content in neutral soil was more abundant than that in saline-alkali soil, which is beneficial to the reproduction of *K. variicola*, the colonization number of *K. variicola* increased rapidly after 6 d, and the final level of colonization exceeded that obtained in saline-alkali soil.

### Effects of *K. variicola* on the rhizosphere soil of maize seedlings

#### Effects of *K. variicola* on organic matter content

**Figure 3:**
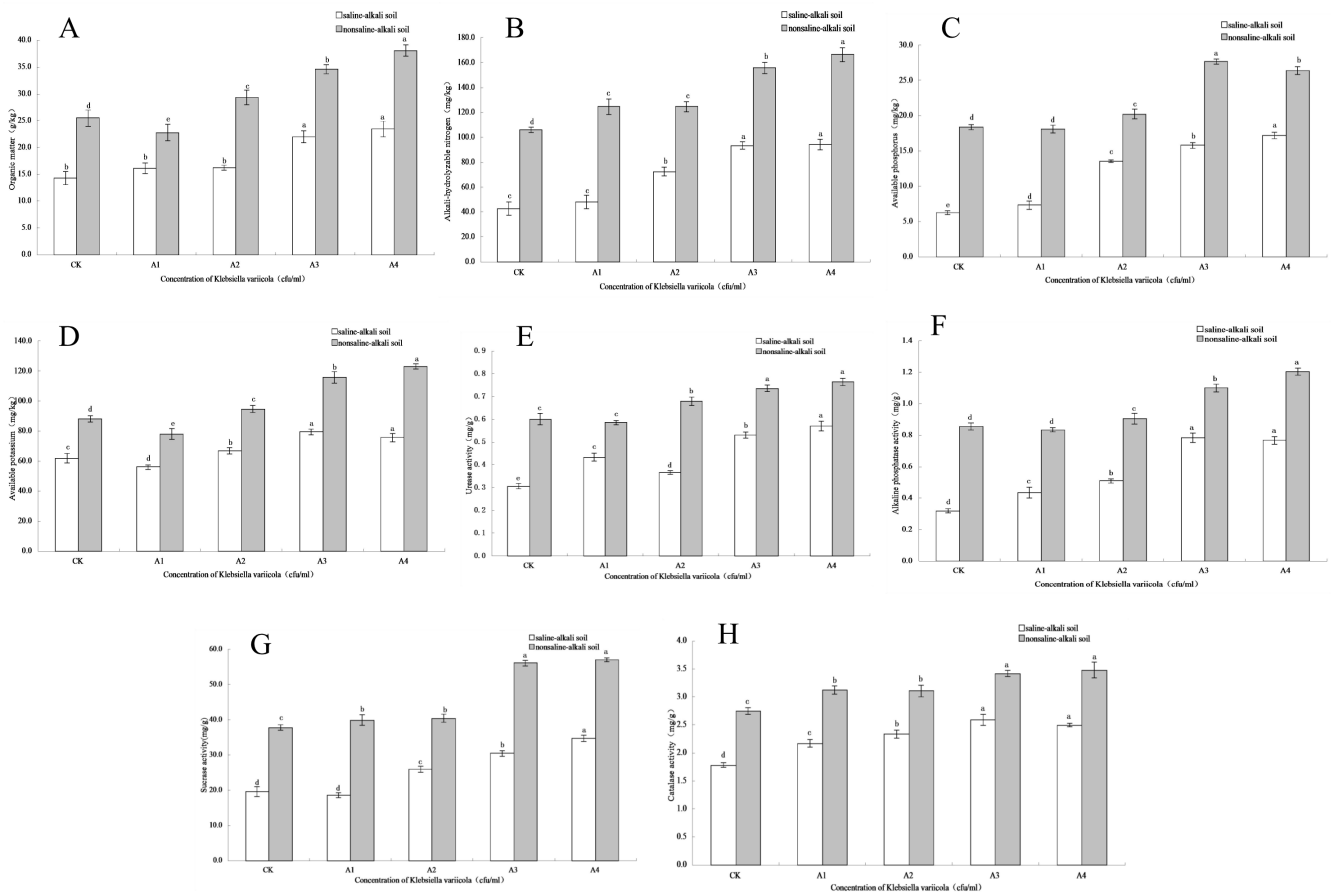
Effects of *K. variicola* on the rhizosphere soil of maize seedlings. (A) Effect of *Klebsiella variicola* on organic matter content. (B) Effect of *Klebsiella variicola* on alkali-hydrolysable nitrogen content. (C) Effect of *Klebsiella variicola* on available phosphorus content. (D) Effect of *Klebsiella variicola* on available potassium content. (E) Effect of *Klebsiella variicola* on urease activity. (F) Effect of *Klebsiella variicola* on alkaline phosphatase activity. (G) Effect of *Klebsiella variicola* on sucrase activity. (H) Effect of *Klebsiella variicola* on catalase activity. CK is the unadded control group,A1 is 1 × 10^2^, A2 is 1 × 10^4^, A3 is 1 × 10^6^, A4 is 1 × 10^8^ cfu/mL concentration of *Klebsiella variicola*.

The content of soil organic matter is closely related to soil fertility and is also the main source of soil nitrogen and phosphorus, which provides energy for crop growth and soil microbial life activities. The organic matter content in saline-alkali and neutral soils increased with increasing *K. variicola* concentration. As shown in Fig. 3A, while saline-alkali stress (CK) significantly decreased (*P* < 0.05) the organic matter content in the rhizosphere of maize seedlings, no significant change was observed in the organic matter content of the rhizosphere of seedlings treated with low concentrations of *K. variicola* (A1 and A2). Organic matter content increased significantly when high concentrations of *K. variicola* (A3 and A4) were applied (*P* < 0.05) and was 64.22% higher in A4 compared to that in CK. In neutral soil, the organic matter content in A1 was lower than that in CK, and significantly increased with the increase in *K. variicola* concentration (*P* < 0.05). The A4 treatment produced the most significant result, which was 49.12% higher than the CK treatment. Owing to the variety of growth-promoting characteristics and products of *K. variicola*, the microbial community structure in the soil was improved, thereby increasing the soil organic matter content. The rate of increase of organic matter content in saline-alkali soil was higher than that observed in neutral soil, possibly owing to poor soil nutrition in, and greater stress on, the rhizosphere microbial community in saline-alkali soils. The effect of saline-alkali stress on the rhizosphere microenvironment of maize seedlings was alleviated by applying *K. variicola*, and the rate of increase of organic matter content in the rhizosphere soil was higher in saline-alkali soil. The results showed that *K. variicola* significantly increased (*P* < 0.05) organic matter content in the rhizosphere soil of maize seedlings, especially in saline-alkali soil, and played an effective role in promoting the growth of maize seedlings in a saline-alkali environment.

#### Effects of *K. variicola* on the content of alkali-hydrolysable nitrogen

Nitrogen is an element present in high amounts in the body, and is a component of many important compounds, including proteins, nucleic acids, chlorophyll, enzymes, and vitamins. The alkali-hydrolysable nitrogen content can reflect the recent supply of soil nitrogen and displays a certain correlation with crop growth. The alkali-hydrolysable nitrogen content in saline-alkali and neutral soils increased with the increase in concentration of applied *K. variicola*. As shown in Fig. 3B, saline-alkali stress (CK treatment) significantly decreased (*P* < 0.05) the alkali-hydrolysable nitrogen content in the rhizosphere soil of maize seedlings. Compared with the CK group, the content of alkali-hydrolysable nitrogen content in the rhizosphere soil increased significantly in all groups except A1 (*P* < 0.05). Once the concentration had reached that of the A3 treatment group, the alkali-hydrolysable nitrogen content did not significantly increase with increases in the applied concentration of *K. variicola*. Compared with the CK treatment, A3 treatment increased by 117.39%. In neutral soil, the alkali-hydrolysable nitrogen content increased significantly with the increase of *K. variicola*, with no significant difference detected between groups A1 and A2. The increase in group A4 was the highest, at 57.32% higher than that in group CK. The results showed that the addition of different concentrations of *K. variicola* could significantly increase the alkali-hydrolysable nitrogen content in maize seedling rhizosphere soil, and that the most effective outcomes in saline-alkali and neutral soils were obtained with the A3 and A4 treatments, respectively.

#### Effects of *K. variicola* on the content of available phosphorus

The content of soil available phosphorus reflects the supply of soil phosphorus, which is a major aspect of the maize growing process in saline-alkali environments. Available phosphorus content was lower in saline-alkali soil. Following the application of *K. variicola*, the available phosphorus content in the soil was significantly increased in all groups (*P* < 0.05). The effect of the A4 treatment was the most significant (*P* < 0.05), at 175.64% higher than that for the CK group (Fig. 3C). As *K. variicola* plays a major role in dissolving phosphorus, the large amount of inorganic phosphorus in the rhizosphere soil of maize seedlings can be fully utilized, thereby significantly increasing (*P* < 0.05) available phosphorus. In neutral soil, the available phosphorus content was highest under the A3 treatment, at 50.74% higher than that of the CK group. The change in available phosphorus content was different under the low concentration treatment, and although there was no significant difference between A1 and CK, a significant difference (*P* < 0.05) was observed between A2 and CK. The results showed that *K. variicola* could significantly increase the available phosphorus content in rhizosphere soil of maize seedlings, especially in saline-alkali soil. This was related to the phosphorus-dissolving function of *K. variicola*.

#### Effects of *K. variicola* on the content of available potassium

Potassium is an essential element for crop growth, and the content of soil available potassium is an important index to estimate potassium supply. The results showed that the application of a high concentration of *K. variicola* in saline-alkali and neutral soils significantly increased (*P* < 0.05) the available potassium content. As shown in Fig. 3D, available potassium content in the rhizosphere soil of maize seedlings under saline-alkali stress (CK) was lower and decreased in the A1 treatment. Subsequently, available potassium content increased significantly (*P* < 0.05) with increasing *K. variicola* concentration. Among the obtained results, available potassium content from the A3 treatment was 28.63% higher than that obtained from the CK treatment. Once the bacterial concentration reached that of the A3 treatment, the available potassium content tended to stabilize, and no significant difference (*P* < 0.05) was observed between A3 and A4. In neutral soil, the A4 treatment was the most effective, with the available potassium content from this treatment being 39.85% higher than that of the CK treatment. The results showed that *K. variicola* could significantly increase the available potassium content in the rhizosphere soil of maize seedlings both in saline-alkali and neutral soils, but that the effect was unsatisfactory at low bacterial concentrations.

#### Effects of *K. variicola* on urease activity

Soil urease activity can be an important index to measure the utilization of nitrogen in soil and plays a major role in improving the saline-alkali tolerance of maize seedlings. As shown in Fig. 3E, saline-alkali stress (CK) significantly decreased urease activity in the maize seedling rhizosphere soil (*P* < 0.05). In saline-alkali soil, the increase in urease activity of *K. variicola* at low concentrations was unstable, with the urease activity of the A2 treatment lower than that of the A1 treatment. Urease activity increased steadily with the increase in applied bacterial concentration, while the highest urease activity was from the A4 treatment, at 86.60% higher than that from the CK treatment. In neutral soil, urease activity from the A1 treatment showed no significant change (*P* < 0.05) compared with the CK treatment; however, while urease activity from the A3 and A4 treatments increased significantly, no significant difference (*P* < 0.05) was detected between these two treatments, indicating that the A3 treatment produced the best application effect in neutral soil, and that any further increase in application rate had no significant effect on urease activity.

#### Effects of *K. variicola* on alkaline phosphatase activity

Soil alkaline phosphatase participates in the decomposition of phosphorus-containing compounds, as well as in the phosphorus cycle, which can be used to characterize the level of soil phosphorus. As shown in Fig. 3F, alkaline phosphatase activity in the rhizosphere soil of maize seedlings significantly decreased (*P* < 0.05) with saline-alkali stress (CK). In saline-alkali soil, alkaline phosphatase activity increased significantly (*P* < 0.05) with increased concentrations of applied *K. variicola* but did not significantly increase (*P* < 0.05) when the concentration reached that of the A3 treatment. No significant difference (*P* < 0.05) was observed between the A3 and A4 treatments. Compared with the CK treatment, the A3 treatment increased alkaline phosphatase activity by 146.08%. There was no significant difference (*P* < 0.05) between the A1 and CK treatments in neutral soil. With the continuous increase in *K. variicola* concentration, the alkaline phosphatase activity increased significantly, and the effect of the A4 treatment was the most significant. Compared with the CK treatment, A4 treatment increased alkaline phosphatase activity by 40.7%. The results showed that *K. variicola* could significantly increase (*P* < 0.05) the activity of alkaline phosphatase in the rhizosphere soil of maize seedlings, both in neutral and alkaline soils, and that the effect was most significant in alkaline soil. Additionally, the application of *K. variicola* could effectively increase the activity of soil phosphatase, promote the transformation and utilization of soil phosphorus, and improve the stress resistance of maize seedlings growing under saline-alkali stress conditions.

#### Effects of *K. variicola* on sucrase activity

Soil sucrase plays a major role in the transformation and utilization of nutrients, as well as and in the carbon cycle, and its activity can reflect the transformation and decomposition of soil organic carbon. In saline-alkali and neutral soils, sucrase activity increased with increasing concentrations of applied *K. variicola*. No significant difference (*P* < 0.05) was detected between the A1 and CK treatments in alkaline soils. With the continuous increase in the concentration of applied *K. variicola*, sucrase activity increased significantly (*P* < 0.05). The A4 treatment was the most effective, where sucrase activity increased by 76.77% compared with CK treatment (Fig. 3G). While different concentrations of *K. variicola* could significantly increase sucrase activity in neutral soil, no significant difference (*P* < 0.05) was observed between the A1 and A2 treatments. The optimal application rate of the A3 treatment increased sucrase activity by 48.47% higher than that of CK treatment, and no significant change in sucrase activity was detected by further increasing the concentration of *K. variicola*. The results showed that the application of *K. variicola* could effectively increase sucrase activity in saline-alkali and neutral soils.

#### Effects of *K. variicola* on catalase activity

Soil catalase can decompose H_2_O_2_ produced as a result of soil biological respiration and biochemical reactions and can alleviate its toxicity to plant roots. As shown in Fig. 3H, catalase activity in the rhizosphere soil of maize seedlings treated with CK was lower in saline-alkali soil. With the increase in *K. variicola* concentration*,* catalase activity in rhizosphere soil of maize seedlings increased significantly (*P* < 0.05). The application rate of the A3 treatment was the most suitable, with catalase activity being 45.29% higher than that of the CK treatment. Compared with CK treatment, different concentrations of *K. variicola* significantly increased (*P* < 0.05) catalase activity in neutral soil, while no significant difference (*P* < 0.05) was detected between the A1 and A2 treatments. The optimal application concentration of the A3 treatment was 24.38% higher than that of CK treatment, and further increases in the applied bacterial concentration produced no significant change (*P* > 0.05) in catalase activity. The results showed that the addition of different concentrations of *K. variicola* in saline-alkali and neutral soils could significantly increase the catalase activity in maize seedling rhizosphere soil, with the A3 treatment being the most suitable.

### Effects of *K. variicola* on the growth of maize seedlings

**Figure 4:**
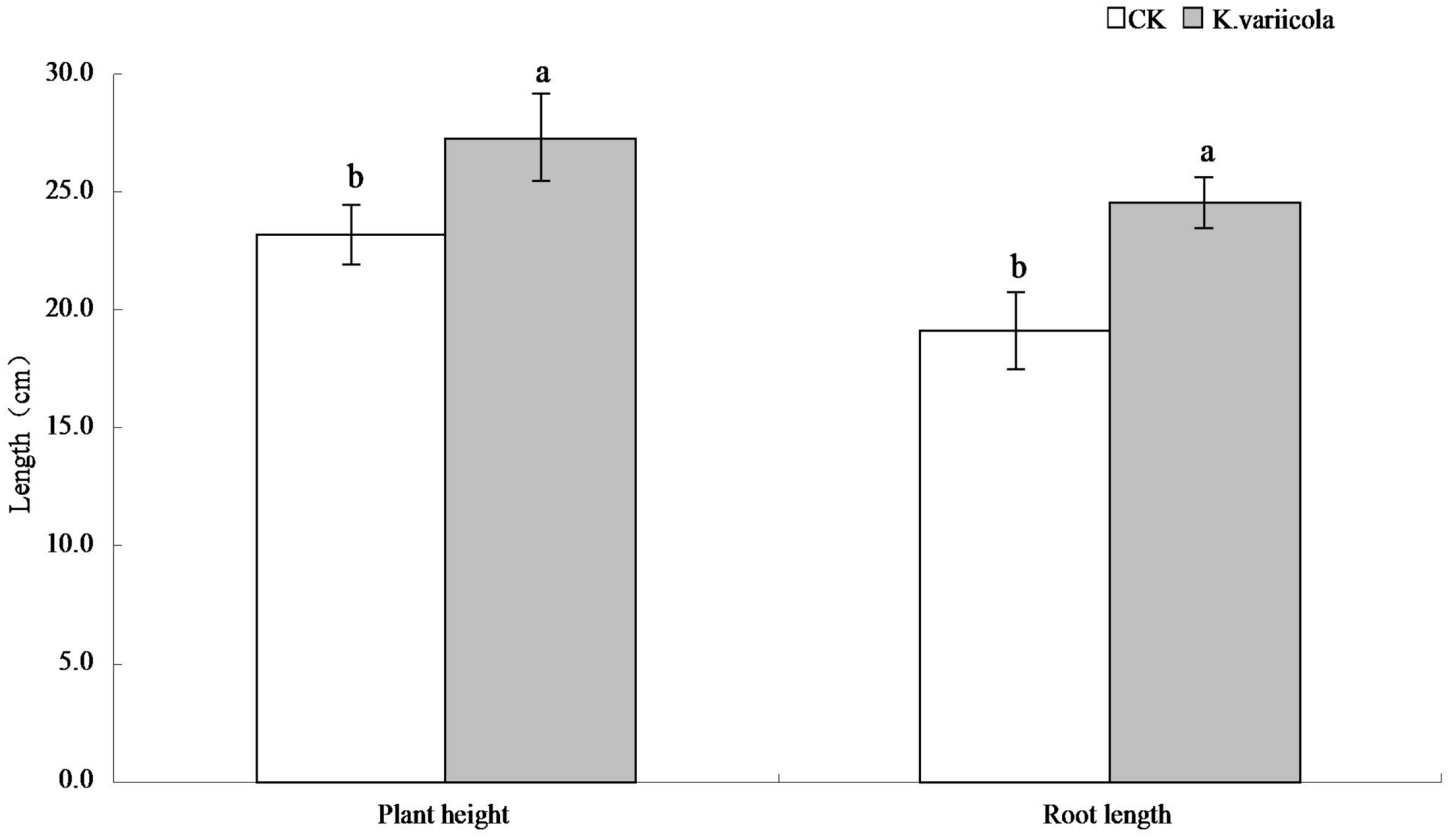
Comparison of maize seedlings with and without *Klebsiella variicola* in saline-alkali soil.

The results of the pot-based experiment showed that *K. variicola* could promote the growth of maize seedlings in neutral soil, as well as improve the saline-alkali tolerance of maize seedlings under saline-alkali stress conditions. In saline-alkaline soil environments, the plant height, root length, as well as aboveground and underground dry weights of maize seedlings were increased by 17.57%, 28.59%, 25%, and 31.48%, respectively, in saline-alkaline soil environments, and by 8.64%, 8.44%, 14.16% and 15.28%, respectively, in neutral soil environments (Table 1). *K. variicola* can therefore effectively promote the growth of maize seedlings, especially under saline-alkali stress conditions (Fig. 4).

**Table 1:** Effect of *Klebsiella variicola* on the growth of maize seedlings effectively promote the growth of maize seedlings.

Concentration (cfu/ml)	Plant height (cm)	Root length (cm)	Ground dry weight (g)	Underground dry weight (g)
SCK	23.22 ± 1.26	19.10 ± 1.66	0.084 ± 0.005	0.054 ± 0.004
SA4	27.30 ± 1.87	24.56 ± 1.09	0.105 ± 0.008	0.071 ± 0.005
NSCK	28.48 ± 1.18	26.29 ± 1.87	0.113 ± 0.009	0.072 ± 0.004
NSA4	30.94 ± 0.84	28.51 ± 1.58	0.129 ± 0.004	0.083 ± 0.008

**Notes.**

SCK is no *Klebsiella variicola* in saline-alkali soil, SA4 is *Klebsiella variicola* in saline-alkali soil, NSCK is no *Klebsiella variicola* in neutral soil, and NSA4 is *Klebsiella variicola* in neutral soil.

## Discussion

### Biological function of *K. variicola*

*K. variicola* can produce indole acetic acid, acetoin, ammonia, phosphorus, and potassium, as well as perform nitrogen fixation, indicating its potential as a type of plant growth-promoting bacteria. This study showed that *K. variicola* can produce indole acetic acid. Following the inoculation of *K. variicola* in liquid medium containing L-tryptophan for 3 d, the concentration of IAA reached 399.43 ± 11.56 mg L^−1^. Related studies have shown that indole acetic acid is one of the important hormones that regulate growth and development processes in plant cells, such as cell enlargement and differentiation, organogenesis, and directional movement (Toscano et al., 2019; Ji, Gururani & Chun, 2014; Timmusk et al., 2014). During the interactions between plants and bacteria, indole acetic acid not only regulates the interaction between bacteria and plants (Duca et al., 2014), it increases plant resistance, and IAA signalling has also been reported to adjust to stomatal opening. Many phytopathogens use open stomata as gateways to enter into the plant, thus by limiting stomatal opening, pathogen entry is also encumbered (Duca et al., 2014). Also, plants with mutations in the IAA biosynthesis pathways have defects in lateral root, root hair and vascular tissue development. Many soil pathogens infect the plant through root tips and lateral root initials. Therefore, changes in plant root architecture or vascular tissue could restrict pathogen entry into the roots and colonization within the plant (Kazan & Manners, 2009; Tsavkelova, Cherdyntseva & Netrusov, 2005), IAA also can be used as a signal molecule improving the balance of indoleacetic indole-acetic acid in host plants by regulating the indoleacetic acid synthesis pathway (Mathesius, 2008; Than et al., 2019). With regards to the bacteria adapting to environmental stress, indole acetic acid can allow the bacteria to better adapt to external stress conditions (150 mmol NaCl) (Bianco et al., 2006; Spaepen, Vanderleyden & Remans, 2007). The ipdC gene in Pa. agglomerans pv. gypsophilae is strongly induced during plant colonization and it allows bacterial cells to colonize in the plant rhizosphere for a long time, in order to alleviate the harm caused by saline-alkali stress to crops. *K. variicola* was cultured in V-P selective medium for 48 h and then mixed with creatine chromogenic solution. The results showed that *K. variicola* produced 152.88 ± 13.56 mg L^−1^ of acetoin. Acetoin is a recognized elicitor and has been proven to not only promote the growth of crops, but also to stimulate plant-induced resistance to infection by *Pseudomonas aeruginosa* in tomato. The research results show that, exogenous application of the B. subtilis derived elicitor, acetoin (3-hydroxy-2-butanone), was found to trigger induced systemic resistance (IsR) and protect plants against DC3000 pathogenesis (Rudrappa et al., 2010). In addition, *K. variicola* can produce large amounts of ammonia. Ammonia played an important role in inhibiting populations of the soybean cyst nematode and southern root-knot nematode. The results showed that ammonia induced the upregulation of 93 disease-resistant genes in soybean and increased the soybean yield by 9.62% (Mi, Marilyn & Ye, 2012). K. variicola grew well on nitrogen-free medium, confirming that *K. variicola* displayed nitrogen-fixing activity. The concentration of soluble nitrogen in the fermentation broth was determined by potassium persulfate oxidation-UV spectrophotometry. The results showed that the total amount of fixed nitrogen reached 15.7 mg L^−1^ in the culture medium, and that nitrogen played an important role in the synthesis of DNA, RNA, protein, and chlorophyll in plants. However, the utilization rate of nitrogen in the agroecosystem was very low. The interaction between non-symbiotic nitrogen-fixing bacteria and plants can provide the latter with available nitrogen as nutrition, and thus, increase plant vegetative growth and increase yield (Elkoca, Kantar & Sahin, 2007; Hayat et al., 2010). Phosphorus is also one of a large number of elements needed for plant growth. K. variicola can degrade inorganic phosphorus and convert insoluble phosphorus in soil, such as phosphate bound with calcium, into a useable form to supply host plants. As for the mechanism of phosphorolysis, there are mainly the following statements. The polysaccharides produced by phosphodiophores and small molecular organic acids can produce acid hydrolysis and complexation to minerals to release phosphate. The secondary metabolites can lead to the change of the microenvironment around mineral particles. Phosphorolytic bacteria can also break the dynamic balance of mineral dissolution and crystallization through the active absorption of K+, thus promoting the release of P and K surrounded by the lattice (Wang, Yuan & Zhao, 2010). Studies have shown that *K. variicola* possesses a variety of biological functions to promote crop growth and is able to extensively colonize the rhizosphere soil of maize seedlings.

### Effect of *K. variicola* on soil nutrient content in the rhizosphere of Maize seedlings

Soil organic matter is an important component of the soil, as well as the main source of various nutrient elements in the soil. It can also adsorb cations in the soil, so that the soil has a certain buffer (Xiang et al., 2020). The content of available nutrients in soil can reflect the short-term supply and release efficiency of soil nutrients. The present study found that the application of *K. variicola* in saline-alkali and neutral soils effectively improved soil nutrition, especially in saline-alkali soil, where the rate of increase was significantly higher than that in neutral soil. Following the application of *K. variicola* in saline-alkali environments, the organic matter, alkali-hydrolysable nitrogen, available phosphorus, and available potassium increased by 64.22%, 117.39%, 175.64%, and 28.63%, respectively. There was a notable increase in alkali-hydrolysable nitrogen and available phosphorus after the application of *K. variicola*, which was consistent with the results of nitrogen fixation and phosphorus dissolution assays with *K. variicola*. The main mechanism of phosphorus-soluble microorganisms is to dissolve insoluble phosphate in soil (Peix, Rivas & Santaregina, 2004). Phosphorus-soluble microorganisms can secrete a lot of organic acids, reduce the pH of their culture medium or soil, and then dissolve phosphate. The results of 4 strains of lysophotic bacteria screened from soybean rhizome showed that strains WJ1, WJ3, WJ5 and WJ6 could secrete a variety of acid substances during their fermentation, among which strains WJ1, WJ3 and WJ6 could produce large amounts of secondary product-ketoglutaric acid (Yang, Wang & Wu, 2016).

### Effect of *K. variicola* on soil enzyme activity in rhizosphere of Maize seedlings

In addition, the results showed that soil enzyme activity in the rhizosphere was significantly altered following the application of *K. variicola*, and that soil enzyme activity played a major role in the transformation and cycling of soil nutrients (Hiradate et al., 2005; Jin et al., 2015). They have important effects on energy transfer, environmental quality, and crop yield (Dick, 1994; Tabatabai, 1994). Soil enzyme activity is greatly influenced by soil organic matter content, which is often used as an indicator to characterize microbial activity and soil fertility (Dick & Tabatabai, 1992; Kumar, Manigundan & Amaresan, 1992). Previous studies have also shown that inoculation of plant growth-promoting bacteria could improve the soil enzyme activity. Some research results showed that inoculation of GM bacteria significantly increased the urease and sucrase activities in upland rice rhizosphere soil (Luo et al., 2015). Other research results show that inoculation strains Glomus Mosseae and Glomus Etunicatum increased the polyphenol oxidase activity of clover rhizosphere soil by 19.6%∼72.0% and 29.7%∼90.6%, respectively, and catalase activity by 3.3%∼12.2% and 7.8%∼34.7%, respectively, indicating an overall increasing trend of acid phosphatase activity (Xiao et al., 2009). The results of this study indicate that saline-alkali stress significantly inhibited the activities of four enzymes in soil; however, these activities gradually increased with increasing concentrations of applied *K. variicola*. The optimum application concentration of *K. variicola* varied for different types of enzymes. At the optimum application rate, alkaline phosphatase activity, sucrase activity, urease activity, and catalase activity increased by 146.08%, 76.77%, 86.60%, and 45.29%, respectively and effectively improved the enzyme activity of maize seedling rhizosphere soil under saline-alkali stress.

### Outlook

In this study, the three-leaf single heart stage, which is the most sensitive to saline-alkali stress in the growth cycle of maize, was taken as the research object. In the future, we will continue to study the effects of *K. variicola* on maize rhizosphere soil at other growth stages. In the future, in-depth studies will be carried out at the molecular level, and combined with transcriptome and metabonomic data, and the effects of *Klebsiella variicola* on maize root metabolism genes and root exudates will be analysed and studied comprehensively on the microenvironment of maize root, root gene expression and interaction mechanisms of root exudates. In addition, this study takes the untreated bacterial liquid as the experimental material, which has higher requirements for the storage conditions of bacteria in agricultural production. In future studies, we intend to develop a protective agent suitable for this strain, to enhance the feasibility of application and distribution.

## Conclusions

We demonstrated that *Klebsiella variicola* possessed a variety of growth-promoting characteristics and were able to extensively colonize in the rhizosphere soil of maize seedlings, thereby significantly improving (*P* < 0.05) the available nutrients and enzyme activities in the rhizosphere soil. *K. variicola* can improve the rhizosphere soil microenvironment of maize seedlings, and consequently promote the growth of maize seedlings, especially under saline-alkali stress conditions. The effects of applying high concentrations of *K. variicola* were better than that of low concentrations, but the effect of improving the soil rhizosphere microenvironment was similar for concentrations of 1 × 10^6^ cfu/ml and 1 × 10^8^ cfu/ml. Considering cost and efficiency, using *K. variicola* concentrations of 1 × 10^6^ cfu/ml may be the most suitable. This study greatly contributes towards the development of a microbial fertilizer suitable for saline-alkali areas, and the exploration of methods that can improve the saline-alkali soil rhizosphere microenvironment and enhance saline-alkali tolerance in maize.

## Supplemental Information

10.7717/peerj.9894/supp-1 Data S1 Soil nutrient data The content of organic matter, alkali-hydrolysable nitrogen, available phosphorus, available potassium.

10.7717/peerj.9894/supp-2 Data S2 Soil enzyme data The content of alkaline phosphatase, sucrase, urease, and catalase

## Additional Information and Declarations

### Data Availability

The following information was supplied regarding data availability: The raw data are available in the Supplemental Files.
